# Direct Bullying and Cyberbullying: Experimental Study of Bystanders’ Motivation to Defend Victims and the Role of Anxiety and Identification With the Bully

**DOI:** 10.3389/fpsyg.2020.616572

**Published:** 2021-01-21

**Authors:** Tomas Jungert, Pinar Karataş, Nathalie Ophelia Iotti, Sean Perrin

**Affiliations:** ^1^ Department of Psychology, Lund University, Lund, Sweden; ^2^ Department of Psychology, Kadir Has University, Istanbul, Turkey

**Keywords:** school bullying, bystanders, prosocial motivation, trait anxiety, depression, identification

## Abstract

School bullying among young adolescents is a globally pervasive problem, but is less common when bystanders are motivated to defend victims. Thus, the focus of this experimental study is on motivation to defend victims of bullying.

**Methods**: A total of 388 students (*M*_age_ = 12.22 years, 49.7% girls) from two Turkish public schools (5th–8th grade) participated in a vignette experiment. Students were randomized to one of two vignettes (direct vs. cyberbullying). Self-report measures of motivation to defend, trait anxiety, depression, and identification with the victim or bully were used.

**Results:** Participants reported more autonomous motivation in the cyberbullying condition, while those who witnessed direct bullying reported higher anxiety and depression.

Results also revealed that this type of condition was associated with anxiety and depression, while anxiety was associated with autonomous motivation to defend. Finally, participants in the direct bullying condition were more likely to identify with the bully.

**Conclusion**: Findings advance our understanding of when and why adolescents are motivated to help victims of bullying because they give a richer picture of what they assess when deciding whether or not they should intervene.

## Introduction

Bullying is a commonly occurring problem for school children globally, with one-year prevalence estimates ranging from 15 to 70% (Hymel and Swearer, 2015). The prevalence of bullying in school appears to be on the rise, with a recent study reporting an increase from 42.7 to 66.4% over 10 years (Waasdorp et al., 2017). Victimization by bullying is now recognized as a risk factor for a wide range of negative health and social outcomes including (among others): lower levels of academic achievement and self-esteem; and higher levels of anxiety, depression, suicidal ideation, and substance use (Reijntjes et al., 2010; Klomek et al., 2013; Landstedt and Persson, 2014; Bjereld et al., 2015; Barzilay et al., 2017). Less is known about the mental health impact of witnessing bullying on children and adolescents. However, studies have found a positive association between depression and anxiety and being a bystander to bullying, albeit less so than for victims of bullying (Juvonen et al., 2003; Glew et al., 2005; Wu et al., 2016).

Bullying is defined in various ways in the literature, but there is a general consensus among researchers that bullying refers to behaviors that harm another person, with intent to do so; the harm may be physical or psychological and is repeated; and there is some kind of power imbalance between the bully and the victim (Farrington, 1993; Olweus, 1993; Rigby, 2002; Sharp and Smith, 2002; Espelage and Swearer Napolitano, 2003; Hymel and Swearer, 2015). Thus, bullying refers to a relationship regarded as continued aggression with a power asymmetry, which can have a significant negative impact on the victim.

Within this broad definition of bullying, it is possible to specify various methods of interaction. According to Farrington (1993), bullying can occur in a face-to-face (direct) encounter between perpetrator and victim or indirectly, either *via* a third party or behind the victim’s back. Direct bullying can involve physical (e.g., hitting and spitting) or verbal (e.g., threats of violence and name calling) attacks on the victim. Indirect bullying may involve spreading rumors about the victim or telling others to exclude the victim from social activities. Today, it often occurs in the form of cyberbullying (Modecki et al., 2014). However it should be noted that, for cyberbullying, the element of repetition is not a fundamental part of the definition, given that one attack can have potentially devastating consequences on the victim, due to a snowball effect, where the effect of a single post/message/picture is amplified throughout the web (Smith et al., 2008; Brighi et al., 2019) Furthermore, the aspect of power imbalance is different because, in cyberbullying, it refers more to a difference in technical abilities with information and communication technologies (ICTs) and to the possibility of anonymity than with an actual or perceived power imbalance between parts (Brighi et al., 2019).

School bullying incidents often have many witnesses and are more frequent in school settings where bystanders reinforce bullying and less frequent when bystanders defend the victims (Salmivalli, 2010; Nocentini et al., 2013). This observation has led to an increase in bullying prevention programs that attempt to increase children’s willingness to intervene on the behalf of bullying victims (Kärnä et al., 2011; Salmivalli et al., 2013) and experimental research to identify factors that influence motivation to defend (e.g., Pozzoli and Gini, 2013). Furthermore, existing studies have found strong positive associations between bullying exposure (witnessing and victimization), depression, and anxiety (Janson and Hazler, 2004; Janson et al., 2009). A study conducted in the United Kingdom predicted that students who witness both direct and indirect bullying risk developing psychological disorders including anxiety and depressive disorders, irrespective of the type of bullying (Rivers et al., 2009).

Bullying *via* mobile phones, the internet, and any kind of electronic communication devices is referred to as cyberbullying and is now recognized as a growing, global problem for children and adolescents (Campbell et al., 2012; Topcu and Erdur-Baker, 2012). For example, Cross et al. (2009) found that among school children aged 11–16 years in the United Kingdom, 33% admitted to cyberbullying someone and 30% reported being a victim of cyberbullying, and Hinduja and Patchin (2012) claim that the prevalence levels of cyberbullying are increasing. Olweus (2012), on the other hand, claims that cyberbullying is an overrated phenomenon when comparing pure cyber-victims and combined victims.

Studies examining young people’s willingness to intervene on behalf of victims of cyberbullying are extremely limited. A recent study by Patterson et al. (2016) found that Australian students (aged 13–16 years) found cyberbullying more dangerous than face-to-face bullying but were less likely to intervene to defend victims of cyber vs. face-to-face bullying. As motivation to defend victims of cyberbullying is less understood than motivation to defend victims of offline bullying, such as a direct and physical type of bullying, the current study aims to investigate if there are differences in response to direct, physical bullying, and indirect, cyberbullying, in terms of motivation to defend, perceptions of dangerousness, and identification with the victim and the bully.

Most bullying includes bystanders who observe the situation. Bystanders’ behaviors can be divided into three categories: reinforcer of the bully, defender of the victim, and outsider (Cowie, 2014). Even though students who are witnesses to bullying find it dangerous and immoral (Wainryb, 2006), observational studies show that bystanders usually choose to reinforce the bully instead of helping or defending the victim (Craig et al., 2000; Lynn Hawkins et al., 2001; Salmivalli et al., 2011). Jennifer and Cowie (2012) found an explanation to this dilemma *via* their study: even if bystanders feel shame and worry, and feel sorry for the victim, their concerns about themselves, fear of personal consequences and of becoming the next target keep them out of helping.

There are many theoretical approaches to explain human motivation in prosocial behaviors. Self-determination theory (SDT) is one theory that has been used recently to explain children’s motivation to defend victims of bullying (Jungert et al., 2016; Iotti et al., 2019; Jungert et al., 2020). SDT explains motivation in a continuum of self-volition, which extends from intrinsic to extrinsic motivation (Ryan and Deci, 2017). Four types of regulations are situated between these two end points; integrated regulation (the most complete form of internalization), identification (when a behavior is regulated by accepting its underlying value), introjected regulation (involves the person’s ego and the emergence of pride or guilt), and external regulation (the classic case where behavior is controlled by external contingencies). In SDT, integrated and identified regulations are considered autonomous motivation, while introjected and external regulations are considered controlled motivation. According to Hardy et al. (2015), people who act prosocially engage in more autonomous motivation.

As stated previously, there is a positive association between the levels of anxiety and depression and having witnessed bullying in youth. This is potentially important in the context of a young person’s motivation to defend victims of bullying because individuals higher in anxiety tend to perceive ambiguous situations as threatening, to exaggerate the potential for harm in threatening situations, and to respond to both ambiguous and threatening situations with higher levels of distress and avoidance (Bar-Haim et al., 2007). Individuals with depression have been found to experience both blunting and exacerbation of the emotional response in stressful situations, but in general to exhibit higher levels of withdrawal or avoidance than individuals who are not depressed (Grillon et al., 2013).

It has been acknowledged in models of bystander behavior, developed primarily with adults in mind, that the emotional state of the witness is likely to exert an influence on their willingness to intervene (Fischer et al., 2011; Hortensius and de Gelder, 2018). For example, a failure to intervene to assist someone in distress has been described as a fear-driven “freezing” or avoidance response that is triggered by high levels of personal distress when other bystanders are present (Hortensius and de Gelder, 2018). By way of contrast, Fischer et al. (2011) have argued that a bystander who perceives the level of danger to the victim (and by extension to themselves) to be high is more likely to intervene. These models make an attempt to address the relationship that state anxiety plays to the bystander effect; however, this relationship has largely remained unexamined in studies of bystander motivation with both adult and child samples. Assuming that a bystander’s levels of state anxiety in bullying situations may exert an influence over their willingness to defend a victim of bullying, it is also reasonable to assume their general or trait level of anxiety is relevant as well. In a previous study (Jungert and Perrin, 2019), it was found that Swedish adolescents with higher levels of trait anxiety were less likely to intervene to defend a victim of bullying, but this was contingent upon the in- vs. out-group status of the victim relative to the bystander. To date no models or studies have examined the link between depression (a trait phenomenon) and bystander motivation in adult or child samples.

There is now a large body of literature which finds that exposure to childhood bullying is associated with an increased risk of mental health problems during childhood and as an adult, particularly (but not limited to) posttraumatic stress, anxiety, and depression (Reijntjes et al., 2010; Copeland et al., 2013; Bannink et al., 2014; Nielsen et al., 2015; Catone et al., 2017). Fewer studies have been carried out to assess the mental health impact of witnessing bullying on children and adolescents, whether offline or online. In addition, what is known largely comes from studies that compare mental health difficulties in bullies vs. victim vs. bystanders. For example, studies have found that students classified as “uninvolved” or as bystanders to the bullying report less depression and anxiety than either victims or bullies (Juvonen et al., 2003; Glew et al., 2005). A population study carried out with 13–15 year olds in Taiwan found that symptoms of social anxiety and depression were positively associated with being a bystander to bullying, albeit less so than for victims, and these symptoms tended to be lower in bystanders who sought to defend the victim compared to those who remained passive (Wu et al., 2016). More recently, research conducted in the United States (Midgett and Doumas, 2019) and Canada (Lambe et al., 2017) indicate that students who observe bullying report experiencing internalizing symptoms, including depression and anxiety. Thus, prior research suggests that being a bystander is associated with anxiety and depressive symptoms. However, to the best of our knowledge, no previous research has investigated whether differences exist in levels of anxiety and depression in youth who have witnessed direct bullying and cyberbullying.

The current research had the following aims: to investigate if different types of bullying (i.e., direct vs. cyber) were associated with different types of bystanders’ motivation to defend victims of bullying; whether anxiety, depression, and perceptions of dangerousness of the bullying situation would mediate the associations between type of bullying and type of motivation to defend, and if witnesses’ identification with the bully and victim would differ between direct and cyberbullying situations. The key dependent variables were: extrinsic motivation to defend, introjected motivation to defend, and autonomous motivation to defend. To measure types of motivation to defend, a sample of participants were presented with either a vignette describing a situation involving direct bullying or a vignette describing indirect cyberbullying.

We hypothesized that cyberbullying would promote greater autonomous motivation to defend than direct bullying (Hypothesis 1a), and that this association in turn would be mediated by anxiety and depression (Hypothesis 1b). More specifically, autonomous motivation to defend would be higher in the cyberbullying condition, compared to the direct bullying condition and that anxiety and depression would mediate the relationship. Moreover, we investigated if bystanders of the different types of bullying would identify themselves more or less with the bully and the victim depending on the type of bullying. We hypothesized that cyberbullying would promote greater identification with the bully (Hypothesis 2a) and with the victim (Hypothesis 2b).

## Materials and Methods

### Participants and Procedure

Participants were recruited from six Turkish school classes (5th–8th grade) in two public schools located in Istanbul, which is the biggest city in Turkey. The data collection took place in May 2018.

The study was authorized by the school administration and student consultants for each class. Before the data were collected, consent of actual participants and parents was prosecuted. The experimenter informed participants that participation was voluntary, that they could refuse to participate in the study, and that they could withdraw from study whenever they wished.

Students from two middle schools (*N* = 453) received written invitations and parent/student consent forms, out of which 390 students volunteered to participate and filled out all measures. Two multivariate outliers (i.e., cases with Mahalanobis distance exceeding the critical value) were identified and removed prior to the analysis. The final sample included 388 adolescent students (49.7% girls; *M* = 12.22 years, *SD* = 0.97 years, range: 11–14 years). Participants were in 6th grade (*N* = 130), 7th grade (*N* = 168), and 8th grade (*N* = 90). All of the participants reported being of Turkish origin. Socio-economic status was not directly measured, but the public schools in Istanbul from which the sample was drawn has students from all socioeconomic backgrounds.

The study was approved by the internal ethics review board at the Department of Psychology, Lund University. Students and their parents were made aware that their participation was voluntary and their responses anonymous, and both had to give active consent to participate.

### Design

The current study utilized an experimental design to test the effect of the type of bullying (direct vs. cyber) on motivation to defend victims. The dependent variables were extrinsic motivation, introjected motivation, and autonomous motivation to defend victims of bullying. The participants filled out paper and pencil questionnaires (anonymously) during class time. The researcher visited each class to explain the purpose of the study and the questionnaire and was available to answer any questions regarding scale items.

Half of the participants were randomized so that they first completed the Revised Children’s Anxiety and Depression Scale (RCADS), then read the vignette, and finally completed the Motivation to Defend Scale (MDS). The other half read the vignette first and then completed the MDS and RCADS. Manipulation checks were used, consisting of one question on the content of the vignette. All participants answered the question correctly.

### Materials

The two vignettes had identical descriptions of a bullying situation except for how direct or cyber the bullying was depicted. The participants were asked to imagine that they were in their schoolyard and witnessed everything that happened in the vignette. In the first condition, the bullying was direct and, in the second condition, the bullying was cyber. The vignettes were about 200 words long (see Appendix).

### Measures

#### Motivation to Defend Scale

The Motivation to Defend Scale (MDS; Jungert et al., 2016) was used to assess early adolescents’ motivation to defend victims during bullying episodes. The items measure four motivational aspects in four subscales: extrinsic, introjected, identified, and intrinsic motivation. This scale was translated into Turkish with back-to-back translation. The scale measures students’ motivation to intervene and defend the victim portrayed in the vignette. Students were asked to indicate “why they would help the victim in the bullying situation.” The scale consisted of five subscales measuring amotivation (two items), extrinsic motivation (four items), introjected motivation (three items), identified motivation (three items), and intrinsic motivation (three items). Example items are “I would not, because I really feel that it is not my responsibility” (amotivation), “To be praised by a teacher” (extrinsic), “To avoid feeling guilty” (introjected), “Because I am the kind of kid who cares about others” (identified), and “Because I like to help other people” (intrinsic). Participants selected an answer that ranged from 1 (“*Totally disagree*”) to 5 (“*Totally agree*”).

In this study, autonomous motivation was calculated as the average of intrinsic and identified regulation, which is a prevalent practice in SDT research (e.g., see Brunet et al., 2015), while introjected motivation and extrinsic motivation to defend were treated as separate variables because of reliability issues. The scales had acceptable reliability: Extrinsic (*ω* = 0.67), Introjected motivation (*ω* = 0.76), and Autonomous motivation (*ω* = 0.65).

#### Revised Children’s Anxiety and Depression Scale

The Turkish version of the Revised Children’s Anxiety and Depression Scale (RCADS; Gormez et al., 2017) is a self-report scale used to assess anxiety and depression in children and adolescents (Chorpita et al., 2000). The RCADS consists of 47 questions assessing symptoms of DSM-IV (Frances et al., 1995) anxiety disorders (generalized anxiety, social phobia, panic disorder, separation anxiety, and obsessive-compulsive disorders) and major depression. The scale was used for assessing the target group’s level of anxiety and depression. For each statement, participants responded along a five-point scale of agreement (1 = *Completely disagree*, 5 = *Completely agree*).

The McDonald’s ω values for all subscales were acceptable: general anxiety disorder (GAD) was 0.79, separation anxiety disorder (SAD) was 0.75, panic disorder (PD) was 0.84, social phobia (SP) was 0.84, obsessive-compulsive disorder (OCD) was 0.74, all anxiety scores was 0.94, and major depressive disorder (MDD) was 0.85. In this study, all anxiety scores and the MDD scale were used.

#### Dangerousness and Identifications

Finally, all participants were asked how much they found the situation in the vignette to be dangerous; how much they identified themselves with the bully and how much they identified themselves with the victim of the vignette. For each statement, participants responded along a 10-point scale of agreement (1 = *Not at all*, 10 = *Totally*). The identification items were transformed into dichotomous variables (quite dangerous/not very dangerous; high degree of identification with the bully and the victim/low degree of identification with the bully and the victim respectively) in order to conduct chi square tests.

### Strategy of Analysis

To investigate if different types of bullying (i.e., direct vs. cyber) were associated with different types of bystanders’ motivation to defend victims of bullying and whether anxiety, depression, and perceptions of dangerousness of the bullying situation would mediate said associations, we tested effects in multiple (parallel) mediator models. Separate analyses were conducted for autonomous, introjected, and extrinsic motivation to defend as the dependent variables. Types of bullying (direct vs. cyber) were used as the independent variables. Bootstrapping with the number of bootstrap samples set at 5,000 was used to calculate 95% confidence intervals for the specific indirect effects. Preacher and Hayes (2008) recommend bootstrapping, especially for testing mediation, because it does not require the normality of the sampling distribution. Independent samples *t*-tests were conducted to investigate differences in the motivation to defend between bystanders of cyberbullying and traditional bullying. To investigate if a type of bullying would promote different identification with the bully, a chi-square analysis was conducted. Jamovi was used in all analyses.

## Results

Table 1 presents the correlations between types of bullying (direct and cyber), motivation (autonomous, introjected, and extrinsic motivation), generalized anxiety, and other related factors. The correlations between all variables were in the small to large range. The correlations between gender and anxiety, major depression, and extrinsic motivation indicated that girls had higher anxiety and depression levels and lower extrinsic motivation than boys. Perceptions of dangerousness in the bullying situation correlated with anxiety, depression, autonomous motivation, and identification with the victim. As expected, the condition correlated positively with autonomous motivation to defend, indicating that autonomous motivation was higher in the cyberbullying condition, but the condition did not correlate with other types of motivation to defend. Surprisingly, the condition also correlated with anxiety and depression, which indicated that anxiety and depression was higher among participants in the direct bullying condition. Moreover, autonomous motivation was significantly correlated with introjected motivation to defend, extrinsic motivation to defend, and anxiety. There were moderate correlations between identification with the bully and extrinsic motivation, anxiety, and depression, while identification with the victim correlated with anxiety and depression, see Table 1.

**Table 1:** Correlations between all variables.

Variable	1	2	3	4	5	6	7	8	9	10	11
1. Gender	-	0.03	−0.10^*^	−0.02^1^	−0.06	−0.04	0.16^**^	−0.18^***^	−0.14^**^	0.04^1^	0.05^1^
2. Age		-	−0.05	0.05	0.04	−0.05	0.01	0.02	0.10^*^	−0.01	0.04
3. Dangerousness			-	−0.03	0.12^*^	−0.02	−0.01	0.25^***^	0.11^**^	−0.01	0.22^***^
4. Manipulation				-	−0.11^*^	−0.01	−0.04	−0.12^*^	−0.14^**^	−0.09^1^	−0.05^1^
5. Autonomous Motivation					-	0.33^***^	−0.34^***^	0.14^**^	0.06	0.01	0.11^*^
6. Introjected Motivation						-	−0.03	0.07	0.05	−0.02	0.09
7. Extrinsic Motivation							-	−0.04	−0.02	0.24^***^	−0.05
8. Anxiety								-	0.74^***^	0.24^***^	0.22^***^
9. Major Depression									-	0.23^***^	0.23^***^
10. Identify with bully										-	0.22^***^
11. Identify with victim											-

1 Spearman’s rho.

*** *p* < 0.001;

** *p* < 0.01;

* *p* < 0.05.

### Impact of Type of Bullying

In line with the hypothesis, independent samples *t*-tests revealed a significant difference in autonomous motivation to defend, whereby those who witnessed cyberbullying reported significantly higher autonomous motivation to defend compared to direct bullying, *t*(385) = −2.20, *p* = 0.028, Cohen’s *D* = −0.22. There was no significant difference between the two conditions and the other types of motivation. However, there was a significant difference in anxiety, whereby those who witnessed direct bullying reported significantly higher anxiety compared to cyberbullying, Welch’s *t*(379) = 2.25, *p* = 0.025, Cohen’s D = 0.23. Finally, depression was significantly higher in students who witnessed direct bullying compared to cyberbullying, Welch’s *t*(373) = 2.77, *p* = 0.006, Cohen’s *D* = 0.28 (see Table 2).

**Table 2:** Means and SDs of the variables in the two conditions.

Variable	Direct *M* (SD)	Indirect *M* (SD)
Autonomous motivation	3.61 (0.82)	3.80 (0.88)^*^
Introjected motivation	4.38 (1.07)	4.36 (0.92)
Extrinsic motivation	2.35 (0.99)	2.27 (1.01)
Anxiety	40.67 (37.00)	35.96 (33.00)^*^
Major depression	9.57 (9.00)	7.83 (7.00)^**^
Perceived dangerousness	4.56 (2.70)	4.42 (2.53)

** *p* < 0.01;

* *p* < 0.05.

### The Mediational Effect of Anxiety, Depression, and Perceived Dangerousness

**Figure 1:**
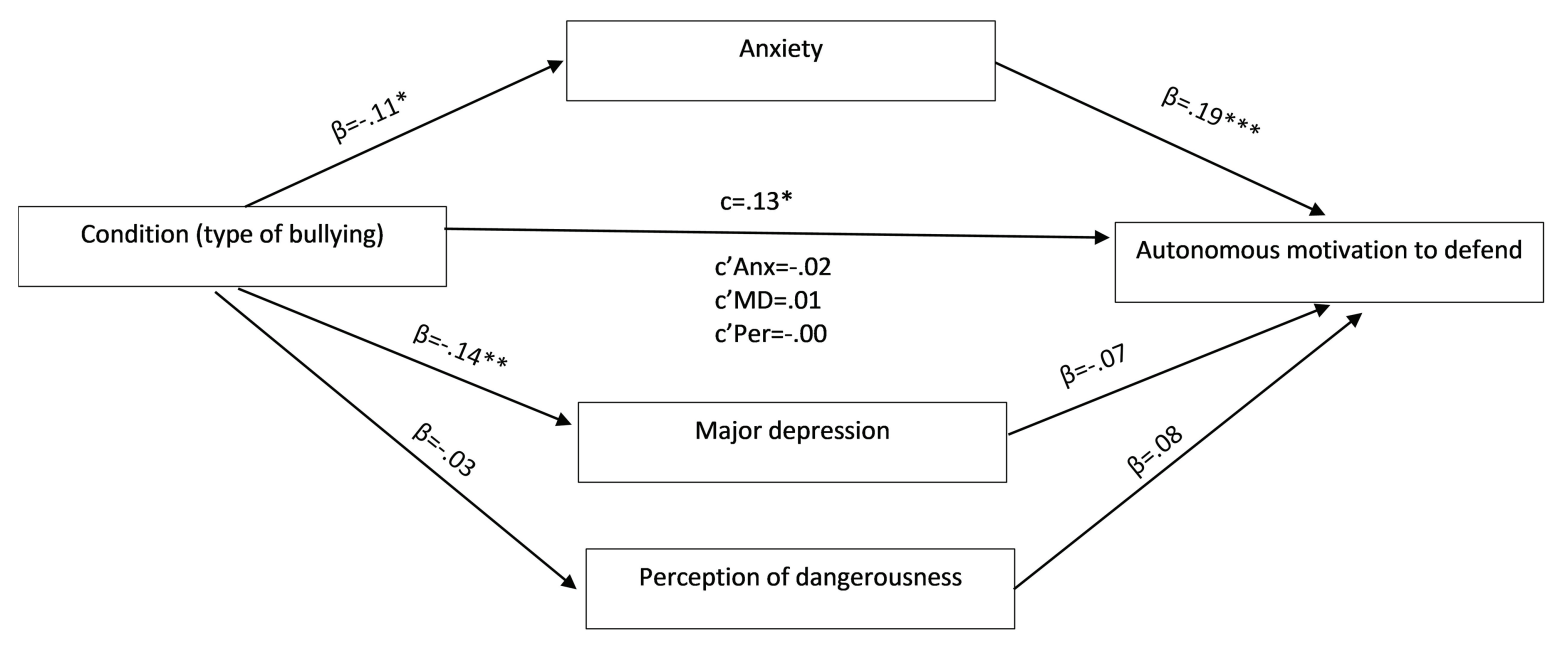
Model of anxiety, depression, danger perception and the relationship between condition (direct/indirect/bullying) and autonomous motivation to defend. Standard error (SE) in parenthesis.

Results revealed that anxiety (*β* = 0.19, *p* < 0.001) was significantly associated with autonomous motivation to defend. Moreover, as predicted, type of bullying was associated with autonomous motivation to defend (*β* = 0.13, *p* = 0.013). In addition, type of condition was significantly associated with anxiety (*β* = −0.11, *p* = 0.029) and depression *β* = −0.14, *p* = 0.007). However, anxiety, depression, and perceived dangerousness did not mediate the effect of type of bullying on autonomous motivation to defend (see Figure 1).

In the mediation models on introjected motivation and extrinsic motivation, no association was significant except for the associations between condition and anxiety and depression as in the model of autonomous motivation to defend (Figure 1).

### Identification With Bully and Victim in Types of Bullying

Table 3 presents the percentages of participants who identified with the bully and with the victim in the two types of bullying situations. The results did not support the hypotheses. Chi-squared analysis revealed that those in the direct bullying condition were significantly more likely to identify with the bully compared to the group in the cyberbullying condition, *χ* ^2^(1, *N* = 387) = 4.00, *p* = 0.046. Chi-squared analysis did not reveal that participants identified themselves more with the victim in the direct bullying condition, *χ* ^2^(1, *N* = 380) = 2.96, *p* = 0.085.

**Table 3:** Percentages of participants who identified with the bully and the victim in the direct condition and the indirect bullying condition.

Variables	Condition	
	Direct bullying	Indirect bullying
Identify with bully	25.9%	17.5%
Identify with victim	51.1%	42.3%

*N* = 380–387.

## Discussion

The aim of the study was to investigate whether direct bullying or cyberbullying would promote higher autonomous motivation to defend the victim, and if anxiety and depression would mediate said association. In line with hypothesis 1a, autonomous motivation to defend was significantly stronger in the cyberbullying condition than in the direct bullying condition. Anxiety was significantly associated with autonomous motivation to defend, but there was no mediating relationship between condition (direct or cyberbullying) and autonomous motivation. An explanation for this result could be that the associations are more direct between type of bullying and autonomous motivation to defend, as well as the associations between anxiety and autonomous motivation to defend. Autonomous motivation to defend plays an important role when witnessing indirect school bullying as well as cyberbullying, when the witness has a higher level of anxiety, which may explain the lack of mediation in the model. Hypothesis 1b was thus not supported. In other words, neither anxiety nor major depression helps describe how or why cyberbullying was associated with higher autonomous motivation to defend. Direct bullying was associated with higher levels of major depression, but depression does not seem to be related to autonomous motivation to defend neither directly or indirectly. In addition, direct bullying was associated with higher levels of anxiety too, but does not seem to be an intermediary variable that could describe the process through which type of bullying is related to motivation to defend. To conclude this, Turkish school children who in our study witness cyberbullying tend to have higher autonomous motivation to defend the victim, but this association is not explained by the mediating influence of having higher levels of depression and anxiety.

A second aim of this study was to explore if there would be differences in identifications with the bully and the victim between conditions. Contrary to the hypotheses (2a and 2b), bystanders identified themselves more often with both the bully and the victim in the direct bullying condition than in the cyberbullying condition. This could be due to the fact that the situation described in the direct bullying vignette may be interpreted as kids just fooling around or playing, which might make it easier for a witness to identify with the involved adolescents, while the situation depicted in the cyberbullying vignette has a character that makes it less easy for the witnesses to identify with the involved peers.

A key aim of the research was to move beyond measuring bystander intentions, by investigating if motivation to defend would differ depending on the type of bullying and if anxiety, depression, and perceptions of dangerousness would be mediating variables. Crucially, analyses revealed that bystanders reported higher autonomous motivation to defend when they witnessed cyberbullying and the association between both conditions and anxiety was positively related to autonomous motivation. This finding can be related to prior research that found being a witness of school bullying is associated with anxiety and depression (Wu et al., 2016; Lambe et al., 2017; Midgett and Doumas, 2019). Results of the current study extend this research by establishing that not only is observing bullying associated with anxiety, but also that direct bulling is associated with higher levels of such internalizing symptoms. These findings add to the research suggesting that the negative consequences of bullying extend beyond students directly involved to witnesses of bullying. Interestingly, we found an association between anxiety and autonomous motivation to defend. Prior studies have demonstrated that the emotional state of witnesses can influence on their willingness to intervene (Fischer et al., 2011; Hortensius and de Gelder, 2018). By contrast, Jungert and Perrin (2019) found that Swedish adolescents with higher levels of trait anxiety were less likely to defend a victim of bullying belonging to an out-group. Results of this study extend this research by demonstrating that state anxiety plays a role in the bystander effect and is associated both with type of bullying and motivation to defend the victim. Thus, bystanders of bullying seem to have a well-integrated set of values when the victim is bullied *via* indirect means such as in cyberbullying. SDT provides an explanation as to why individuals are autonomously motivated to help victims of school bullying (Ryan and Deci, 2017). One possible explanation could be that children and adolescents today find it more meaningful and fun to intervene when they observe indirect bullying, which often occurs in the form of cyberbullying, where they also might feel more self-efficacious. On the other hand, we found that the bystanders more often identified themselves with both the victim and the bully in the direct bullying condition. The identification with the bully in the direct bullying conditions may explain why autonomous motivation to defend the victim was lower in that condition. Identifying with the bully hints that participants define to a lower extent the direct bullying scenario as bullying. Boulton et al. (2002) found that adolescents are less likely to include behaviors that they engage in themselves in their definition of bullying. Thus, the observers who in the current study identified themselves with the bully may have perceived the vignette as aggressive non-bullying behavior, which would not trigger any kind of motivation to defend victims. The indirect cyberbullying condition, however, involved relational bullying in which the victimization was aimed at damaging the peer relationships of the victim, which may fall into the observers’ definition of bullying more readily, and may explain the higher autonomous motivation to defend the victim.

Taken together, these findings indicate that adolescents are more likely to help a victim of cyberbullying because they like to help and think it is important to help under such circumstances, while they find it easier to identify with both the victim and the bully in direct bullying. Thus, the bystander effect plays an important role, as the type of bullying determines how strong the autonomous motivation to help is, and that neither perception of dangerousness nor identification with the victim strengthens motivation to defend, but that it is rather the type of bullying that has the greatest impact on motivation to defend victims.

Our study helps to put a focus on the bystanders who are often overlooked, even though they have a lot of power in preventing the occurrence of bullying (Salmivalli, 2014). Prevention programs might do better if they first assess the extent to which any individual student perceives the type of bullying. The intervention might help the child to become aware of how the various types of bullying influence motivation. In line with what Monks and Smith (2006) suggest, it seems important that clear definitions of bullying are used and that anti-bullying programs emphasize that bullying should be distinguished from fighting. Furthermore, adolescents need to be assisted to recognize the consequences, not only of their own aggressive actions, but also of aggressive actions by their peers, in order to increase their autonomous motivation to defend victims in direct and cyberbullying alike.

## Limitations and Future Directions

While the study benefitted from a large sample size, experimental methods, and the use of standardized measures, certain limitations need to be noted. First, all data was collected *via* questionnaire, thus there is a risk for common method variance (Podsakoff et al., 2012). Second, while we tried to eliminate social desirability through the use of anonymous surveys, it cannot be entirely ruled out that this presentation phenomenon influenced our results. Third, predictors of bystander motivation and not actual bystanding behavior were the focus in the present study. Even if previous studies on prosocial interventions have shown that intentions powerfully indicate real behavior (Smith and McSweeney, 2007), further studies are needed involving mixed methodologies, including observational designs and peer nominations (Morcillo et al., 2015) and findings may differ across alternative intergroup contexts such as ethnicity (Abbott and Cameron, 2014; Mulvey et al., 2014). Therefore, further studies are needed in other countries, and involving a more diverse range of ethnic groups.

## Conclusion

The current study demonstrates that cyberbullying elicits stronger autonomous motivation to defend victims in adolescent bystanders compared to situations of direct bullying, and that adolescents identify themselves more with bullies and victims in direct bullying situations than in cyberbullying. Taken together, these results advance our understanding of when and why adolescents are motivated to help victims of school bullying because they help us give a clearer picture of what they evaluate when deciding whether or not they should intervene. Future studies should build upon these findings and focus on investigating these associations further, perhaps in a qualitative manner, in order to provide researchers with firsthand accounts of the thought processes that adolescents employ when evaluating their involvement in bullying situations as possible defenders.

## Data Availability Statement

The raw data supporting the conclusions of this article will be made available by the authors, without undue reservation.

## Ethics Statement

The studies involving human participants were reviewed and approved by the internal ethics review board at the Department of Psychology, Lund University. Written informed consent to participate in this study was provided by the participants’ legal guardian/next of kin.

## Author Contributions

TJ was the principal investigator with overall responsibility for all aspects of the study. PK collected the data and with NI and SP contributed to the design of the study and preparation of the article. All authors approved the submitted version.

### Conflict of Interest

The authors declare that the research was conducted in the absence of any commercial or financial relationships that could be construed as a potential conflict of interest.

## APPENDIX

### Traditional Bullying

Kim is a new student at your school. The first day at school, the “cool” gang are openly looking at each other and laughing and making faces when Kim is introduced to the class. Kim gets both sad and angry when noticing the reactions of the “cool” gang, but tries to hide those feelings as much as possible and is properly introduced. After this, the teacher tries to make a statement to the gang, but they do not seem to care. This was Kim’s first day at school, and since then, Kims situation has not improved. Rather, it has deteriorated. The “cool” gang continues teasing Kim and making nasty jokes without the teacher knowing about it, as they do it mostly during recess when no teacher is around. One day during recess, Kim is standing alone in the schoolyard; the others are in groups, talking and holding their cell phones, while no one is looking at Kim. Suddenly, one kid from the “cool” gang walks up to and takes a picture of Kim who is completely unprepared. The person says that they will make the image more interesting by changing it and threatens to upload it to Snapchat, so that everyone at school can see the ugly nerd who is new in their class. Kim is your classmate and you are standing with your friends just a few yards away. You have heard everything that has been said. You see that Kim is scared, sad, pale and avoiding eye contact, and seems to have completely frozen.

### Cyber Bullying

Kim is a new student at your school. The first day at school, things seem to go well when Kim is introduced to the class. However, there is a gang that are whispering and fiddling with their cell phones while Kim is being introduced to the class. Kim gets both sad and angry when noticing the reactions of the “cool” gang, but tries to hide those feelings as much as possible and is properly introduced. The teacher does not seem to pay any attention to this. This was Kim’s first day at school. The following weeks, you see pictures that are posted with comments about Kim on social media and the comments seem to get worse. One day, Kim approaches some kids at school that seem to be nice, but they all look the other way upon noticing that Kim is walking up to them. Kim gets sad, gives up and walks over to a bench and is now sitting there alone. Kim can see how other students in various groups are fiddling with their cell phones, snickering as they look between their screens and Kim. They look at each other as if something funny is going on. Kim is your classmate and you know that Kim has noticed the mean posts and comments on internet and cell phones. You see that everyone ignores Kims own comments. Kim is scared, sad, pale and avoiding eye contact, and seems to have completely frozen.
